# The Effects of a 12-Month Weight Loss Intervention on Cognitive Outcomes in Adults with Overweight and Obesity

**DOI:** 10.3390/nu12102988

**Published:** 2020-09-29

**Authors:** Jamie C. Peven, John M. Jakicic, Renee J. Rogers, Alina Lesnovskaya, Kirk I. Erickson, Chaeryon Kang, Xueping Zhou, Alexis Porter, Shannon D. Donofry, Jennifer C. Watt, Chelsea M. Stillman

**Affiliations:** 1 Department of Psychology, University of Pittsburgh, Pittsburgh, PA 15260, USA; lesnovskaya@pitt.edu (A.L.); kiericks@pitt.edu (K.I.E.); jcwatt@pitt.edu (J.C.W.); cstillmancoyne@gmail.com (C.M.S.); 2 Center for the Neural Basis of Cognition, Carnegie Mellon University, Pittsburgh, PA 15213, USA; 3 Healthy Lifestyle Institute, University of Pittsburgh, Pittsburgh, PA 15260, USA; jjakicic@pitt.edu (J.M.J.); rjrogers@pitt.edu (R.J.R.); 4 College of Science, Health, Engineering and Education, Murdoch University, Perth, WA 6150, Australia; 5 Department of Biostatistics, University of Pittsburgh, Pittsburgh, PA 15260, USA; crkang@pitt.edu (C.K.); xuepingzhou@pitt.edu (X.Z.); 6 Department of Psychology, Carnegie Mellon University, Pittsburgh, PA 15213, USA; alexisp@andrew.cmu.edu; 7 Department of Psychiatry, University of Pittsburgh, Pittsburgh, PA 15260, USA; sdd14@pitt.edu

**Keywords:** exercise, weight loss, reward sensitivity, executive function

## Abstract

Obesity is associated with poorer executive functioning and reward sensitivity. Yet, we know very little about whether weight loss through diet and/or increased exercise engagement improves cognitive function. This study evaluated whether weight loss following a dietary and exercise intervention was associated with improved cognitive performance. We enrolled 125 middle-aged adults with overweight and obesity (98 female) into a 12-month behavioral weight loss intervention. Participants were assigned to one of three groups: energy-restricted diet alone, an energy-restricted diet plus 150 min of moderate intensity exercise per week or an energy restricted diet plus 250 min of exercise per week. All participants completed tests measuring executive functioning and/or reward sensitivity, including the Iowa Gambling Task (IGT). Following the intervention, weight significantly decreased in all groups. A MANCOVA controlling for age, sex and race revealed a significant multivariate effect of group on cognitive changes. Post-hoc ANCOVAs revealed a Group × Time interaction only on IGT reward sensitivity, such that the high exercise group improved their performance relative to the other two intervention groups. Post-hoc ANCOVAs also revealed a main effect of Time, independent of intervention group, on IGT net payoff score. Changes in weight were not associated with other changes in cognitive performance. Engaging in a high amount of exercise improved reward sensitivity above and beyond weight loss alone. This suggests that there is additional benefit to adding exercise into behavioral weight loss regimens on executive functioning, even without additional benefit to weight loss.

## 1. Introduction

More than one third of the U.S. population meets criteria for obesity (i.e., a body mass index (BMI) ≥ 30.0 kilograms/meter^2^ (kg/m^2^)) and approximately 68% of adults in the U.S. are considered overweight (BMI ≥ 25.0 kg/m^2^) [1]. Obesity increases risk for numerous diseases including heart disease, Type II diabetes, and cerebrovascular disease [2,3]. Obesity has also been consistently linked to deficits in cognitive and brain health outcomes [4,5,6]. Particularly in the context of executive functions (e.g., inhibitory control, set-shifting, working memory, decision-making), higher BMI is associated with poorer task performance regardless of whether the individual meets criteria for obesity [7]. This suggests that even small to modest amounts of extra weight could negatively impact executive processes critical to regulating health behaviors, including diet and exercise, which may exacerbate the vicious cycle of weight gain and, in turn, limit weight loss or weight loss maintenance.

At present, the majority of the published studies in this field are cross-sectional and do not target the mechanisms underlying these associations. While the mechanisms of how excess weight is related to cognitive processes are not entirely clear, they may include metabolic dysfunction [8,9], chronic inflammation [10], or influences on neural morphology and function such as reduced dendritic spine density, gray matter volume, or activation in the prefrontal cortex—a region critical for supporting executive functions [11,12]. Garnering an understanding of whether changes in weight positively influence cognitive functioning is critical for developing successful interventions to improve brain health and determine whether weight-related brain deficits can be remediated.

In addition to cognitive deficits, obesity is also associated with poorer performance on reward processing tasks, especially those involving food-related cues [13,14]. Specifically, individuals with obesity attend longer to food-related cues [15,16] and are more motivated to eat following exposure to food cues [17] compared with healthy-weight counterparts. Further, participants with obesity also show altered reward processing for non-food stimuli [18,19], indicating that reward processing and reduced executive function may be closely linked. Yet, we have a poor understanding of whether weight and impairments in reward processing are causally related to one another. Work to date has been largely cross-sectional and has not addressed causal directionality or the modifiability of these associations. Specifically, it is not clear whether weight-related deficits in reward responses or executive functioning can be mitigated or remediated by weight loss.

There is inconsistent evidence for the efficacy of weight loss interventions to improve cognitive functioning and, further, whether the addition of exercise to a weight loss regimen could further alter these effects. Interventions that have shown cognitive improvements following weight loss suggest that losing weight may improve certain cognitive domains (i.e., executive functions, attention, memory) more than others [20,21,22,23]. For example, greater weight loss following a diet and behavioral weight management intervention was associated with better performance on executive functioning tasks, such as the Stroop task [21], or tasks requiring both executive functioning and reward processing, such as the Iowa Gambling Task [23]. In contrast, several behavioral weight loss interventions have failed to show improvements in cognitive task performance relative to control groups [24,25]. However, despite demonstrating weight loss, most of the interventions failing to show cognitive effects have been short-term (i.e., six months or less). It is possible that greater success in losing weight and maintaining weight loss through longer-term behavioral interventions is related to their demonstrated cognitive benefits [26]. Thus, it may be that only sufficiently long interventions can both aid weight loss and improve cognitive outcomes. Since there is strong evidence that exercise can improve cognition [27,28], combining diet and exercise in a behavioral weight loss intervention might provide additive benefits to cognitive health outcomes [29]. However, few studies have examined the effects of exercise on the relationship between weight loss and cognitive functioning beyond the effects of weight loss through diet alone.

Exercise, even in the absence of significant weight loss, is an effective, non-pharmacologic method to improve cognitive functioning across multiple domains [30]. Numerous meta-analyses have demonstrated that older adults in exercise intervention groups show improvements in cognitive functioning, including executive functions, relative to non-exercising controls [21,27,30]. Although much of the research in this area has been conducted in older adults, one recent randomized clinical trial of aerobic exercise demonstrated that six months of activity improved executive functioning in adults aged between 20–67 years [28]. Notably, these studies have all been conducted without examining or controlling for weight loss, which may contribute its own unique benefits to cognition. Thus, there is a need to investigate how long-term and multi-modal weight loss interventions influence cognitive function and whether weight loss, in conjunction with exercise, may provide additive benefits.

The primary aim of this ancillary study was to determine if a 12-month randomized clinical trial (RCT) of an energy-restricted diet and prescribed exercise altered executive functioning and reward responsivity in adults with overweight and obesity. Further, we evaluated whether exercise, in combination with an energy-restricted diet, could improve cognitive functioning beyond weight loss through diet alone (i.e., a dose-response effect of exercise). To accomplish these aims, we measured cognitive function in adults with overweight or obesity using a battery of reward and executive functioning tasks both before and immediately after 12 months of a behavioral weight loss intervention involving: (1) diet modification only, (2) diet plus 150 min per week of exercise, or (3) diet plus 250 min per week of exercise. First, we predicted that the intervention would improve performance on the reward-related and executive functioning tasks regardless of group. Second, we predicted a dose-response effect of exercise, such that intervention effects on cognitive functioning would be greatest in the group receiving the most exercise, followed by the moderate exercise group, and that the smallest effects would be observed in the diet-only group.

## 2. Materials & Methods

Details of this intervention have been previously described [31] and will be reviewed briefly here.

### 2.1. Participants

One hundred twenty-five participants (98 female) were recruited from a randomized clinical trial (ClinicalTrials.gov NCT01500356; R01HL103646; PI: Jakicic) with the primary outcome to examine the effects of weight loss and exercise on measures of cardiac magnetic resonance imaging (MRI). Details about participant recruitment for this trial can be found in Rogers et al. (2019) [32]. Participants volunteered to enroll in an ancillary study examining the effects of weight loss and exercise on cognitive and brain health (R01 DK095172; PI: Erickson). Participants who volunteered for this ancillary study were between the ages of 18–55 (44.63 ± 8.36 years) with a BMI ranging from 25.0–39.9 kg/m^2^ (32.22 ± 3.96 kg/m^2^). As previously reported [32], participants were excluded from the parent study for the following reasons: (1) age < 18 or > 55 years; (2) BMI < 25.0 or >40.0 kg/m^2^; (3) self-reporting ≥60 min per week of structured moderate-to-vigorous intensity physical activity (PA); (4) weight loss of ≥5% or participation in a weight-reduction diet within the prior six months or a history of bariatric surgery; (5) presence or history of cardiometabolic disease (e.g., hypertension, congestive heart failure, diabetes mellitus) or cancer; (6) taking medication that could affect heart rate or blood pressure; (7) taking medication that could influence body weight; (8) treatment for psychological conditions that included medication or counseling, including eating disorders; (9) currently pregnant, pregnant within the prior six months, or planning a pregnancy within the next 12 months; (10) contraindication to MRI; (11) current alcohol or substance abuse; (12) planning on geographical relocation outside of the region within 12 months or inability to attend at least 80% of the scheduled intervention sessions; or (13) inability to comply with the components of the intervention. Additionally, participants were ineligible for this ancillary study for the following reasons: (1) presence or history of a neurological disorder (e.g., dementia, stroke), traumatic brain injury, or developmental pathology; (2) left-handedness. Individuals with a history of welding work or tattoos with metal filings were subject to additional MRI safety screening prior to participation in both the parent study and this ancillary study.

Eligible participants provided written informed consent and completed brain MRI and cognitive testing sessions within 30 days of beginning and immediately following the 12-month intervention. The cognitive data are the focus of the present study. This study was approved by the University of Pittsburgh Institutional Review Board (PRO12030272) in accordance with the Declaration of Helsinki.

### 2.2. Physiological Outcomes Testing

#### 2.2.1. Weight Assessment

Weight was assessed in duplicate on a calibrated digital scale (Tanita Digital Scale, Model #WB-110A) to the nearest 0.1 kg and height was measured in duplicate on a calibrated wall-mounted stadiometer (Perspective Enterprises, Inc., Portage, MI, USA) at baseline and then again at months six and 12 of the intervention. Objective weight and height measurements were used to calculate BMI (kg/m^2^).

#### 2.2.2. Cognitive Assessment

Tasks measuring executive functioning as well as non-food-related reward processing were administered at baseline and post-intervention. Change scores were created for all cognitive variables of interest by subtracting baseline from post-intervention.

##### N-Back

N-back tasks measure working memory function by having participants determine whether a target letter presented on a screen matches a letter seen in a previous trial. This version of the N-back task was administered as a blocked design that incorporated alternating 1- and 2-back conditions. In the 1-back condition, participants were asked to identify whether the target letter on the screen matched or did not match the letter presented in the preceding trial. In the 2-back condition, they were asked to identify whether the target letter on the screen matched or did not match the letter presented two trials earlier. In all conditions, participants were asked to respond by pressing a button when they saw a target letter and another button when the letter presented was not a target. In each block, a series of 16 letters were presented in white font on a black background for 1.5 seconds (1-second inter-stimulus interval). There were six blocks (three 1-back, three 2-back) for a total of 48 trials per condition. Reaction times (RT), percentage of correct responses, and errors were recorded for each condition. A working memory difference score was calculated in which RT during the trials in the 1-back condition was subtracted from the 2-back condition, reflecting the cost associated with more challenging working memory conditions. Here, a higher score indicates worse performance with longer RT during the more difficult 2-back condition. The main outcome metric for analyses was a change in this working memory difference score, with lower scores indicating a greater improvement in performance from baseline to follow-up.

##### Task Switch

Task switching requires participants to alternate between rules either between or within blocks of task trials. There were two single-task conditions with each single-task presented in separate blocks of trials. In one of the single-task conditions, participants determined whether a number was greater than or less than the number 5 by pressing the letter “m” or “n” with their index and middle fingers on their preferred hand on a standard keyboard. In the second single-task condition, participants determined whether a number was even or odd by pressing the letter “m” or “n” with their index and middle fingers on their preferred hand on a standard keyboard. Participants were instructed to respond as quickly and accurately as possible. In the switch-task condition, participants alternated between the previously described single-tasks within the same block of trials. In the present study, participants completed a 32-trial block of each single-task condition (two single-task blocks in total), as well as one 64-trial block of the switch-task condition for a total of 128 trials. The main outcomes were mixing cost, calculated as the difference between the average RT during single-task blocks and the average RT during mixed blocks, and switching cost, calculated as the difference between switch RT and repeat RT within the mixed blocks [33]. Mixing cost reflects the costs associated with the more challenging working memory condition, while switching cost reflects the costs associated with repeating versus alternating task conditions in sequential trials. For both metrics, higher costs suggest poorer performance.

##### Stroop Color-Word Task

Participants completed a color-word Stroop task to measure inhibitory control. Participants completed 182 trials of the task in the MRI and wore a glove on their right hand with buttons to indicate a response choice corresponding with each finger. During the Stroop task, they were asked to indicate the color of the text that was written on the screen, regardless of the word that was written. In the congruent condition, the word presented matched the color of the text (e.g., “red” written in red ink). In the incongruent condition, the word presented did not match the color of the text (e.g., “red” written in green ink). In the neutral condition, the word presented was not a color name (e.g., “table” written in red ink). Comparison of the RT during the incongruent and congruent conditions can be quantified as the Stroop effect, a metric of interference that measures inhibitory control [Van der Elst, 2006 #67} and was used as the main outcome of the task. The equation used to calculate the Stroop effect was: (incongruent RT–congruent RT)/congruent RT. Higher Stroop effect scores are indicative of greater interference or poorer performance, on the task.

##### Iowa Gambling Task (IGT)

A computerized version of the Iowa Gambling Task (IGT) [34] was administered. At the beginning of the task, participants were given a $2000 credit with which they were instructed to play a game with the goal of winning as much money as possible. They were instructed to select cards one at a time from one of four decks (i.e., Deck A, B, C or D) presented on the screen. They were told that some decks were better than others, but they were not told which decks were better. Participants were also told that the game was fair, so they should play as if they were using real money. Participants completed five blocks of 20 card selection trials, totaling 100 trials. There were two main outcome variables from the IGT task. The most common outcome is the net payoff score (“p”), which reflects long-term tracking of rewards in the task; the other (“q”) is thought to reflect more immediate sensitivity to previous losses [35]. The net payoff score (p) was calculated by subtracting the number of cards chosen from disadvantageous decks from the number of cards chosen from advantageous decks. The sensitivity to frequency of reward and punishment score (q) was calculated by subtracting choices from the high loss frequency decks from choices from low loss frequency decks. For both p and q, higher scores are indicative of better performance.

#### 2.2.3. Intervention Groups

After completing baseline testing, participants were randomly assigned to one of three weight loss interventions. All three intervention conditions involved participants attending in-person group sessions and individual telephone sessions that focused on behavioral strategies to assist engagement in the prescribed behaviors aimed to facilitate weight loss. Group sessions were conducted separately for each intervention condition to minimize potential contamination and sessions were scheduled weekly for the first 24 weeks and then approximately every other week for weeks 25–52 [31]. Each session was scheduled for 30–60 min. If an in-person group session was missed, an attempt was made to conduct an individual make-up session. Individual telephone calls were scheduled for approximately every other week, which corresponded to weeks when an individual session was not scheduled, during the latter half of the intervention (i.e., weeks 25–52). These telephone sessions were approximately 10 min in duration and followed a script to address key aspects of the intervention, focusing on continued engagement in the key elements of the intervention (diet or diet plus PA). Additional key elements of each intervention condition are described briefly below.

##### Diet-Only (DIET)

Participants randomized to DIET were prescribed only an energy restricted diet [31]. Energy intake was prescribed at 1200–1800 kilocalories per day (kcal/day) based on baseline body weight (<200 pounds (lbs) = 1200 kcal/day; 200 to 250 lbs = 1500 kcal/day; >250 lbs = 1800 kcal/day) and height. To facilitate the adoption of the dietary recommendations and to provide guidance on meal options and portion sizes, participants were provided with example meal plans that were designed by a registered dietician. Participants were permitted to self-select their food options and were given a calorie counter book as a reference to facilitate adjustment of portion sizes based on their selections. They were instructed to record their food choices and portion sizes in a diary that was given to them by the investigators. They returned these diaries to the intervention staff for review at each in-person intervention session. The intervention staff provided written comments on the diaries to assist the participants in adjusting their dietary choices in a manner that would facilitate weight loss or weight loss maintenance.

##### Diet + Moderate Exercise (DIET + MODEX)

Participants in DIET + MODEX received the dietary intervention as described for the DIET intervention. In addition, DIET + MODEX was prescribed exercise that progressed from 100 min per week to 150 min per week by week 9 of the intervention [31]. 150 min of weekly exercise were prescribed in the parent study to reflect the public health guidelines for engaging in physical activity [36]. Participants engaged in this exercise remotely and were not monitored objectively during the activity. Participants were encouraged to engage in exercise on five days per week to achieve their weekly prescribed goal; however, they could choose their exercise days each week to accommodate their individual schedules. They were instructed to exercise for at least 10 min during each session that counted toward their weekly goal. Exercise during those sessions was instructed to be at a moderate-to-vigorous intensity, which was self-monitored using a rating of perceived exertion scale. To achieve this prescribed intensity, participants were encouraged to engage in cardiovascular activity, such as brisk walking or other similar intensity activities (e.g., cycling) and the specific mode of exercise was self-selected. Participants were instructed to record their exercise in a diary that was given to them by the investigators. As with the food diaries, the participants returned these diaries to the intervention staff for review at each in-person intervention session. The intervention staff provided written comments on the diaries to assist the participant in adjusting their exercise and overcoming barriers in an effort to maximize engagement in the prescribed amount of exercise.

##### Diet + High Exercise (DIET + HIGHEX)

Participants in DIET + HIGHEX received the dietary intervention as described for the DIET intervention. In addition, the DIET + HIGHEX group was prescribed exercise that progressed from 100 min per week to 250 min per week by week 25 of the intervention [31]. 250 min of weekly exercise reflect weight loss guidelines suggesting that 250 weekly minutes may be needed to enhance long-term weight loss and minimize weight gain [36]. All other aspects of the exercise intervention for DIET + HIGHEX were consistent with what is described above for DIET + MODEX.

### 2.3. Statistical Analyses

We used an intent-to-treat approach for all described analyses. All participants were invited to complete follow-up assessments, regardless of their enrollment status in the study (i.e., enrolled or withdrawn) or adherence to the intervention. All participants who enrolled in this ancillary study completed the follow-up assessments and so all were included in the current analyses. Bivariate Pearson’s product moment correlations revealed significant associations between several demographic characteristics and individual cognitive change scores. Thus, age, sex and racial group (i.e., white versus non-white) were included as covariates in all analyses. All outcome variables (i.e., weight and cognitive change) were checked for normal distribution prior to entry into any analyses. Since all variables were normally distributed, no additional data transformation was performed.

#### 2.3.1. Intervention Effects

Mixed-effects analysis of variance (ANOVA) assessed the effectiveness of the intervention on the primary physiological outcome: weight. Group (DIET, DIET + MODEX, DIET + HIGHEX) was included as a between-subjects factor and Time (baseline, follow-up) was the repeated measures factor. The same analysis was run with exercise as the outcome measure to confirm that participants were adhering to the intervention protocol. Details about intervention-related weight change for this subsample of participants in this ancillary study have been previously reported [31].

#### 2.3.2. Cognitive Effects

To examine the cognitive effects of the intervention and to reduce the chances of Type I error from multiple comparisons, we conducted a multivariate analysis of covariance (MANCOVA) using the change scores from each cognitive task as dependent variables and age and sex as covariates. We then decomposed any omnibus effect from the MANCOVA with separate analyses of covariance (ANCOVAs) on the individual tasks. These individual ANCOVAs were also used to assess the effectiveness of the intervention or the main effect of Time.

## 3. Results

### 3.1. Participants

Of the 125 participants who volunteered to enroll in the ancillary study, the DIET + MODEX group was smaller in size (*n* = 30) compared to the DIET + HIGHEX (*n* = 45) and DIET (*n* = 50) groups. However, the intervention groups were well-matched on key demographic characteristics (all *p*-values > 0.44) (Table 1). Overall, participants were middle aged (44.38 ± 8.59 years) and highly educated (16.37 ± 2.67 years). 98 of the 125 (78.4%) were female and 91 (72.8%) were White. Participants’ average BMI at the time of enrollment was 32.44 ± 3.93 kg/m^2^, which met criteria for obesity. Additional information about participants’ change in self-reported exercise at the end of the intervention has been previously published [31].

### 3.2. Missing Data

Of the 125 participants initially enrolled in the study, nine participants did not complete the intervention (lost to follow-up per group: DIET *n* = 4, DIET + MODEX *n* = 2, DIET + HIGHEX *n* = 3). Thus, 116 participants completed all follow-up assessments. A chi-square test of those who completed versus did not complete the follow-up assessments was not significant (*p* = 0.800), indicating that group assignment did not influence the proportion of missing data. A comparison of those who completed the intervention versus those who did not revealed that completers attained greater education (16.54 ± 2.53 years) than non-completers (14.22 ± 3.49 years), *F* (1,121) = 6.569, *p* = 0.012. However, no other demographic differences were seen for age, sex, race, or baseline BMI. The overall lack of group differences on demographic characteristics suggests that these data are likely missing at random. However, we cannot rule out the possibility that other unmeasured characteristics related to the ability and motivation to maintain exercise are missing not at random.

### 3.3. Intervention Effects

#### 3.3.1. Weight Loss

**Figure 1:**
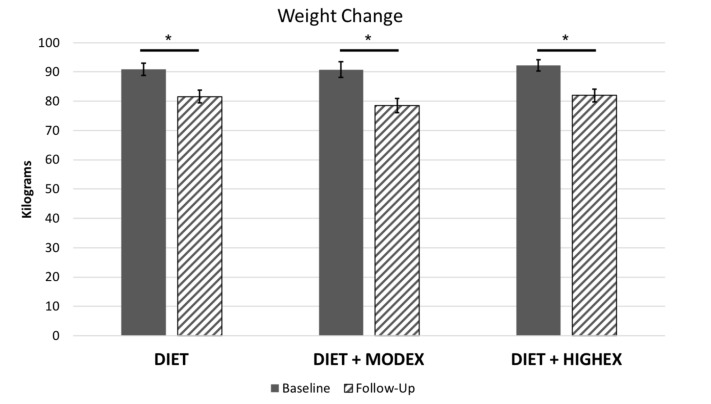
Intervention effects on weight (kg), separated by group assignment. Error bars represent standard error. Note: * indicates that the comparison was significant at the *p* < 0.05 threshold.

There was a main effect of Time on weight, *F* (1,112) = 186.40, *p* < 0.001, indicating that the intervention was effective at reducing weight across all groups (Figure 1), with an average weight loss of 9.93 ± 7.79 kg. There was no evidence that weight loss was moderated by group assignment, as there was not a significant Time × Group interaction (*p* = 0.363). Similarly, there was a significant reduction in BMI across all participants (loss of 3.50 kg/m^2^) that also was not moderated by group assignment (*p* = 0.292). Additional information about changes in weight and adherence to the intervention for this subsample of participants who participated in this ancillary study have been previously reported [31].

#### 3.3.2. Executive Functioning

Participants’ baseline executive functioning scores were not significantly different as a function of intervention group (all *p*-values > 0.05, see Supplementary Materials Tables S1–S4 for additional information). The MANCOVA adjusted for age, sex, and racial group and examined the effect of group assignment on cognitive changes. In line with our hypotheses, the MANCOVA showed a significant multivariate effect of Group (DIET + MODEX, DIET + HIGHEX or DIET) on cognitive change (Pillai’s Trace = 0.20, *F* (4,106) = 1.925, *p* = 0.033), indicating that cognitive changes were dependent on group assignment and suggests a dose-response effect of exercise. Table 2 includes details of the specific cognitive variables entered into the MANCOVA, which include interference scores (see Supplementary Materials Tables S1–S4 for all task scores across all groups). An additional MANCOVA including the change scores of only the most cognitively-demanding task conditions (i.e., Task Switch switching, N-back 2-back, Stroop incongruent) was run to confirm that any significant changes were not due to improvements in easier task conditions (i.e., Task Switch repeating, N-back 1-back, Stroop congruent). The results of this MANCOVA did not differ from the analysis that included interference scores, and so the original MANCOVA was further evaluated.

**Figure 2:**
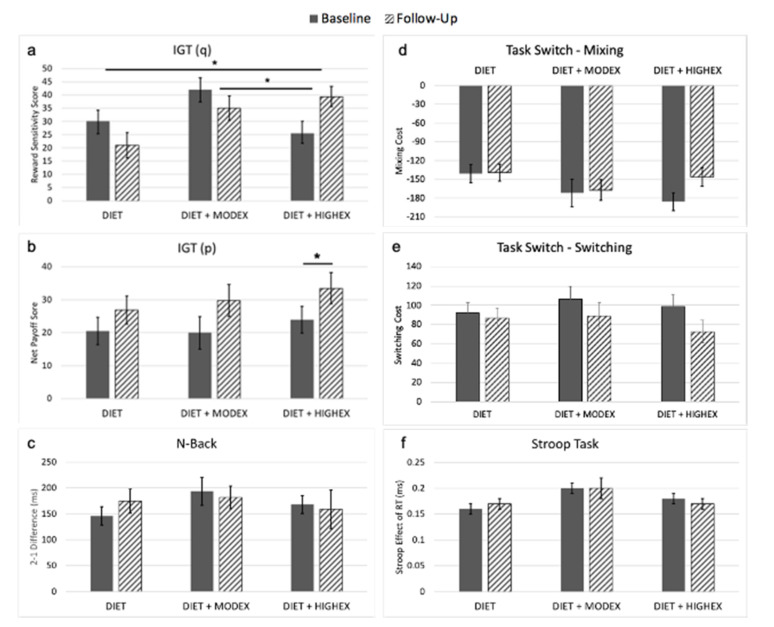
Cognitive performance for each group before and following the intervention. Error bars represent standard error. (**a**–**f**) show the individual cognitive change from baseline to follow-up assessments. Note: * indicates that the *post-hoc* comparison was significant at the *p* < 0.05 threshold.

*Post-hoc* univariate ANCOVA analyses controlling for age, sex, and racial group revealed that this MANCOVA effect was driven by the IGT reward frequency sensitivity, as there was a significant Group × Time interaction (*F* (2,114) = 6.813, *p* = 0.002; Figure 2a). Consistent with our hypotheses, the nature of this univariate interaction was such that the group randomized to the highest amount of prescribed exercise showed significantly greater improvements on reward processing compared to the other intervention groups. That is, the DIET + HIGHEX group showed improvements in the IGT sensitivity score relative to the DIET + MODEX and DIET groups (all *p*-values ≤ 0.004), while both the DIET + MODEX and DIET groups’ scores declined. However, there were no significant differences between the DIET and DIET + MODEX groups. While no significant Group × Time interactions were detected for the other tasks (all *p*-values > 0.230), we observed a main effect of Time on IGT net payoff score (*F* (1,112) = 6.126, *p* = 0.015) and Task Switch mixing cost (*F* (1,110) = 5.878, *p* = 0.017), which appear to be driven by the DIET + HIGHEX group (Table 2, Figure 2b,d,e).

Bivariate correlations revealed no relationships between weight change and change in any cognitive task score (all *p*-values > 0.175). Therefore, no additional analyses were conducted investigating weight change as an independent predictor.

## 4. Discussion

This study examined the effects of a behavioral weight loss intervention consisting of an energy restricted diet and exercise on cognitive performance in 125 sedentary adults with overweight and obesity. The intervention was effective at reducing weight across all participants, although, in contrast to our hypotheses, there was no moderation by intervention group despite prior evidence and weight loss guidelines indicating a dose-response of exercise [36]. This suggests that participation in prescribed exercise, even at a dose well above the current physical activity guidelines [37], did not increase weight loss above and beyond diet alone. Although group assignment did not moderate weight loss, MANCOVA analyses of the cognitive data revealed an effect of intervention group on cognitive changes. This effect was largely driven by improvements in reward sensitivity, with no significant omnibus changes in performance detected for working memory, task switching, or inhibitory control tasks.

Prior weight loss interventions have shown mixed results regarding the efficacy of weight loss for improving cognitive performance. Despite our hypotheses, the results of our intervention suggest that weight loss alone was not sufficient to induce improvements in executive functions and reward processing, and support those studies that did not show intervention effects on cognition [25,38]. Our findings are consistent with a recent meta-analysis suggesting that executive functions are not among the cognitive processes improved by weight loss [22]. That we were able to detect improvements in task performance, albeit not statistically significant, only for the DIET + HIGHEX group suggests that high levels of prescribed exercise may be a driving factor for cognitive change above and beyond weight loss. Given the inconsistency in previous literature, it is also possible that weight loss interventions that demonstrate cognitive improvements differ in methodology from our study in ways that are critical for the detection of these effects. For instance, interventions that show improvements in cognitive functioning tend to be shorter in duration than our 12-month study. One such intervention demonstrated that greater weight loss over four months was associated with improved executive function (i.e., Stroop task) [39]. It is therefore possible that the reported cognitive improvements from other briefer interventions were related to transient benefits of initial or more rapid weight loss that are not sustained.

Engaging in exercise yields greater cognitive gains over 12 months than weight loss through diet alone, particularly in the context of reward processing. Our findings are consistent with the literature reporting greater improvements in executive functioning relative to other cognitive domains (e.g., language) following exercise interventions in normal-weight participants (e.g., [28,30,40]), and go beyond these earlier studies by evaluating the effects of a multi-modal (i.e., diet plus exercise) intervention including participants with overweight and obesity. Given that individuals with obesity show altered executive control in cross-sectional studies [14], it is important to consider how the addition of exercise improves specific executive functions above and beyond weight loss alone. Here, the addition of 250 min of weekly activity to a caloric-restricted diet (DIET + HIGHEX) resulted in greater improvements in reward sensitivity relative to a group not engaging in any additional exercise (DIET). Of note, however, the DIET + HIGHEX group did not show any additional weight loss beyond the other groups, nor was weight loss correlated with improvements in IGT performance in *post-hoc* analyses. These results suggest that substantial weight loss might not be needed to detect cognitive benefits found with increased exercise.

The only significant improvement in task performance was for the IGT, suggesting that some cognitive processes (i.e., reward processing) might be more sensitive to the effects of exercise than others. This improvement was dose-dependent, which indicates that only higher engagement in exercise improved sensitivity to rewards. This was further reflected in the unanticipated decline in IGT q scores for the DIET + MODEX group, suggesting that the addition of a higher amount of exercise is critical to improving some cognitive functions. Given that there are underlying differences in neural reward processing in individuals with obesity relative to normal-weight controls [41] and between physically inactive versus highly trained athletes [42], it is plausible that exercise engagement may mitigate some maladaptive behavioral reward responsivity. These findings are consistent with literature from both human and animal models demonstrating that engagement in exercise alters neurophysiological responses to rewards (e.g., opioid receptor binding, dopaminergic or glutamatergic signaling, respectively), thereby improving reward sensitivity [43,44]. Importantly, both the DIET + MODEX and DIET groups showed declines in IGT reward sensitivity, although the DIET group declined to a greater extent than the exercise group. It is possible that there is a threshold for exercise to show improvements in reward processing and the DIET + MODEX group did not meet this threshold. With additional exercise, the DIET + MODEX may have achieved a small, yet significant, benefit to reward sensitivity to non-food stimuli. More work must be done to capture whether there is a dose-response of exercise on reward processing in all populations.

This was the first study to evaluate the effects of an exercise intervention on IGT performance. While executive functioning has been frequently studied in the context of exercise interventions, the IGT and its relationship to sensitivity to rewards has not. To date, only one multi-modal intervention has evaluated changes in IGT performance but did not show beneficial effects. Delgado-Rico and colleagues (2012) enrolled 42 adolescents ranging from normal weight to obesity in a 12-week intervention that included exercise, dietary counseling, and cognitive behavioral therapy. After the intervention, participants’ IGT scores did not change, nor were they related to changes in BMI [45]. However, due to the multi-modal nature of this study, it is not possible to determine the unique influence of exercise on the lack of cognitive change. In contrast to the described study, our findings demonstrate that prescribing 250 min per week of exercise within the context of a behavioral weight loss program is a successful intervention to improve reward sensitivity within the IGT in middle-aged adults.

Improvements in reward processing following a high dose of exercise may have clinical implications for sustaining weight loss after an intervention. Reduced sensitivity to high frequency rewards and losses may allow individuals to be less reactive in everyday life. In fact, some research shows that reduced sensitivity to rewards on the IGT is associated with lower attrition and greater weight loss over the course of a 16-week weight loss intervention [46,47]. Executive processes like planning and inhibitory control may be related to reward sensitivity, which would be beneficial in the context of a behavioral weight loss intervention. Individuals with better executive abilities may be able to adhere to dietary and exercise plans more easily, thus allowing them to continue to achieve their weight loss goals. Given that there were no differences between our intervention groups at baseline, increasing exercise is one way to reduce reactivity to rewards and execute the cognitive processes to complete the intervention successfully. The decline in reward processing for the DIET + MODEX group may, however, have been due to some of the limitations of our study described in detail below.

Contrary to our predictions, there was no statistically significant dose-dependent effect of exercise on working memory, task switching, or inhibitory control tasks, although there was a main effect of Time detected for Task Switch mixing cost and IGT net payoff score. The main effect of Time suggests that, while cognitive performance may have improved for some tasks, the improvements in these cognitive processes were not dependent on exercise engagement. As previously described, it is possible and likely that exercise engagement does not affect all executive functions equally, as it does not uniformly affect all cognitive domains [48,49,50]. At present, the exercise intervention literature is mixed, with some studies showing positive effects of exercise for individual executive processes (e.g., set shifting, working memory, inhibitory control) but not others [51,52,53]. While we demonstrated that engaging in exercise during weight loss has benefits to reward processing during the IGT, other executive processes may not be as sensitive to exercise engagement in overweight and obese populations. Supporting this idea, pairwise comparisons of IGT net payoff score and Task Switch mixing cost between groups suggested that the main effect of Time detected for these tasks was driven by the DIET + HIGHEX group. However, it is also possible that our lack of significant findings was influenced by low statistical power due to small intervention group sample sizes. Future work should explore whether a larger sample size would increase the statistical power sufficiently to detect intervention group differences in executive functioning.

There are several limitations of our study that should be considered in light of our findings. First, although executive functions are known to be sensitive to exercise engagement [30,54], we did not administer a comprehensive neuropsychological assessment to our participants to test the effects of exercise on other cognitive domains. Thus, it is possible that other cognitive domains may also show a similar effect. We chose to focus on executive functions since changes in this domain have been detected in both the weight loss and exercise literatures. However, future studies with more comprehensive cognitive batteries to target domains such as memory and/or processing speed are warranted. Second, 78.4% of our sample was female, limiting the generalizability of our findings to adult males. Third, all participants volunteered for this ancillary study from the larger parent study, which prohibited our ability to balance the three intervention groups. Although participants were randomized to the parent study, recruitment based on volunteering for the ancillary study may have created an unanticipated bias in our sample. Finally, our study did not collect follow-up data beyond the completion of the intervention, limiting our ability to draw long-term conclusions about the maintenance of weight loss or cognitive change.

Despite these limitations, our findings contribute to the field in several important ways. This 12-month weight loss intervention was the first to test the dose-response effects of exercise on executive functioning and reward sensitivity above and beyond the effects of weight loss through diet alone. In a sample of 125 overweight and obese middle-aged adults, our results suggest that engaging in a high amount (i.e., 100 min more than the daily recommendation) of exercise improves executive functioning, specifically sensitivity to rewards as measured by the IGT. This study demonstrates that there is an added benefit to higher volumes of exercise engagement during weight loss when compared with moderate levels of activity or no additional activity, even if there is no additional benefit to weight loss.

## Figures and Tables

**Table 1:** Demographic characteristics of participants by group. Data are presented as mean (standard deviation).

	DIET M (SD)	DIET + MODEX M (SD)	DIET + HIGHEX M (SD)	SIGNIF.
*n*	50	30	45	
Age	43.26 (8.91)	45.70 (7.57)	44.76 (8.88)	0.443
% Female	80.0%	73.3%	80.0%	0.746
% White	72.0%	70.0%	75.56%	0.860
BMI (kg/m^2^)	32.55 (3.51)	32.32 (4.35)	32.39 (4.15)	0.965
Weight (kg)	90.85 (14.74)	90.82 (14.49)	92.27 (13.10)	0.863
Education (years)	16.22 (2.71)	16.45 (2.88)	16.48 (2.78)	0.882

Note: MODEX = moderate exercise intervention group; HIGHEX = high exercise intervention group; SIGNIF. = significance (*p*-value); BMI = body mass index; kg = kilograms; m = meters.

**Table 2:** Cognitive change scores by intervention group. Indicators of significant differences were adjusted for age, sex, and racial group.

	DIET M (SD)	DIET + MODEX M (SD)	DIET + HIGHEX M (SD)
IGT Reward Sensitivity (q) *	−9.87 (36.97)	−9.61 (24.56)	13.98 (31.43)
IGT Payoff (p)	4.61 (31.38)	8.46 (29.87)	10.07 (35.17)
Task Switch Mixing Cost (RT, ms)	6.35 (115.39)	0.76 (114.78)	33.21 (107.35)
Task Switch Switching Cost (RT, ms)	−6.39 (93.90)	−26.71 (91.91)	−35.82 (94.79)
N-Back 2-1 (RT, ms)	27.49 (180.70)	−21.86 (141.92)	−21.51 (225.83)
Stroop Effect (RT, ms)	0.008 (.06)	−0.007 (0.10)	−0.02 (0.08)

* *p* < 0.05 from *post-hoc* comparisons. Note: MODEX = moderate exercise intervention group; HIGHEX = high exercise intervention group; IGT = Iowa Gambling Task; RT = reaction time; ms = milliseconds. Higher IGT reward sensitivity (q) and net payoff (p) scores indicate better performance (i.e., reduced sensitivity or higher score, respectively); lower Task Switch, N-Back, and Stroop Effect scores indicate better performance (i.e., reduction in switch costs or faster RT).

## Supplementary Materials

The following are available online at https://www.mdpi.com/2072-6643/12/10/2988/s1, Table S1: Iowa Gambling Task, Table S2: Task Switching, Table S3: Stroop Task, Table S4: N-Back Task.
