# Supplementary material for: The Effects of a 12-Month Weight Loss Intervention on Cognitive Outcomes in Adults with Overweight and Obesity
Source: Nutrients. 2020 Sep 29;12(10):2988. doi: 10.3390/nu12102988 (PMC7600527; doi:10.3390/nu12102988)
Supplement: Supplementary file 1 [file nutrients-12-02988-s001.pdf]

Supplemental Table 1. Iowa Gambling Task. Pre- and post-intervention group scores are presented as mean (SD).

	**DIET**		**DIET + MODEX**		**DIET + HIGHEX**	
	PRE	*POST*	*PRE*	*POST*	*PRE*	*POST*
**Total Payoff**	20.52 (29.50)	26.91 (28.75)	19.97 (26.84)	29.79 (25.56)	23.87 (27.17)	33.49 (29.94)
**Reward Sensitivity Score**	30.12 (29.94)	21.00 (32.54)	42.10 (24.57)	35.07 (24.63)	25.56 (30.25)	39.39 (24.61)

Note: There were no between-group differences (all *p*-values > 0.05) on any pre-intervention value.

Supplemental Table 2. Task Switching. Pre- and post-intervention group scores are presented as mean (SD).

	DIET		DIET + MODEX		DIET + HIGHEX	
	PRE	*POST*	*PRE*	*POST*	*PRE*	*POST*
**Repeat RT**	862.83 (118.66)	838.78 (119.23)	865.80 (136.51)	846.71 (89.18)	880.23 (89.87)	863.48 (95.08)
**Switching RT**	955.23 (147.62)	925.85 (110.72)	972.06 (152.94)	935.64 (102.32)	979.34 (113.39)	914.50 (158.75)
**Mixing Cost**	-141.13 (102.97)	-139.29 (90.84)	-172.01 (121.11)	-167.24 (86.42)	-185.73 (95.73)	-146.25 (91.35)
**Switching Cost**	92.41 (73.20)	87.07 (67.76)	106.26 (72.85)	88.93 (72.17)	99.11 (79.82)	72.91 (72.37)

Note: There were no between-group differences (all p-values > 0.05) on any pre-intervention value.

Supplemental Table 3. Stroop Task. Pre- and post-intervention group scores are presented as mean (SD). Stroop effect is calculated as (incongruent–congruent)/congruent.

	**DIET**		**DIET + MODEX**		**DIET + HIGHEX**	
	PRE	*POST*	*PRE*	*POST*	*PRE*	*POST*
**Congruent RT**	768.46 (81.33)	754.12 (91.63)	746.30 (84.45)	752.99 (95.75)	769.29 (67.76)	791.86 (104.01)
**Incongruent RT**	891.26 (126.78)	882.85 (142.54)	897.01 (122.86)	907.79 (164.76)	909.55 (104.51)	922.30 (127.47)
**Neutral RT**	795.90 (88.55)	791.81 (104.60)	779.01 (90.51)	786.64 (101.67)	802.92 (68.93)	826.28 (104.21)
**Stroop Effect**	0.16 (0.09)	0.17 (0.09)	0.20 (0.08)	0.20 (0.11)	0.18 (0.08)	0.17 (0.08)

Note: There were no between-group differences (all p-values > 0.05) on any pre-intervention value.

Supplemental Table 4. N-Back Task. Pre- and post-intervention group scores are presented as mean (SD).

	**DIET**		**DIET + MODEX**		**DIET + HIGHEX**	
	PRE	*POST*	*PRE*	*POST*	*PRE*	*POST*
**1-Back RT**	872.10 (152.57)	821.34 (201.01)	884.50 (174.68)	875.33 (167.58)	872.00 (154.87)	821.62 (194.65)
**2-Back RT**	1018.08 (163.14)	996.14 (169.34)	1078.08 (186.05)	1057.37 (192.71)	1040.08 (166.82)	980.45 (195.22)
**2-back – 1-back RT**	145.98 (125.41)	174.80 (156.78)	193.58 (146.53)	182.04 (115.24)	168.07 (117.54)	158.83 (235.94)

Note: There were no between-group differences (all p-values > 0.05) on any pre-intervention value.
